# MSH3 Mismatch Repair Protein Regulates Sensitivity to Cytotoxic Drugs and a Histone Deacetylase Inhibitor in Human Colon Carcinoma Cells

**DOI:** 10.1371/journal.pone.0065369

**Published:** 2013-05-28

**Authors:** Jae Myung Park, Shengbing Huang, David Tougeron, Frank A. Sinicrope

**Affiliations:** 1 Mayo Clinic and Mayo Cancer Center, Rochester, Minnesota, United States of America; 2 Department of Gastroenterology, Poitiers, France; Shanghai Jiao Tong University School of Medicine, China

## Abstract

**Background:**

*MSH3* is a DNA mismatch repair (MMR) gene that undergoes frequent somatic mutation in colorectal cancers (CRCs) with MMR deficiency. MSH3, together with MSH2, forms the MutSβ heteroduplex that interacts with interstrand cross-links induced by drugs such as cisplatin. To date, the impact of MSH3 on chemosensitivity is unknown.

**Methods:**

We utilized isogenic HCT116 (*MLH1−/MSH3−*) cells where *MLH1* is restored by transfer of chromosome 3 (HCT116+ch3) and also *MSH3* by chromosome 5 (HCT116+3+5). We generated HCT116+3+5, SW480 (*MLH1+/MSH3+*) and SW48 (*MLH1−/MSH3+*) cells with shRNA knockdown of *MSH3*. Cells were treated with 5-fluorouracil (5-FU), SN-38, oxaliplatin, or the histone deacetylase (HDAC) inhibitor PCI-24781 and cell viability, clonogenic survival, DNA damage and apoptosis were analyzed.

**Results:**

*MSH3-*deficient *vs* proficient CRC cells showed increased sensitivity to the irinotecan metabolite SN-38 and to oxaliplatin, but not 5-FU, as shown in assays for apoptosis and clonogenic survival. In contrast, suppression of *MLH1* attenuated the cytotoxic effect of 5-FU, but did not alter sensitivity to SN-38 or oxaliplatin. The impact of *MSH3* knockdown on chemosensitivity to SN-38 and oxaliplatin was maintained independent of *MLH1* status. In *MSH3*-deficient *vs* proficient cells, SN-38 and oxaliplatin induced higher levels of phosphorylated histone H2AX and Chk2, and similar results were found in *MLH1*-proficient SW480 cells. *MSH3*-deficient vs proficient cells showed increased 53BP1 nuclear foci after irradiation, suggesting that MSH3 can regulate DNA double strand break (DSB) repair. We then utilized PCI-24781 that interferes with homologous recombination (HR) indicated by a reduction in Rad51 expression. The addition of PCI-24781 to oxaliplatin enhanced cytotoxicity to a greater extent compared to either drug alone.

**Conclusion:**

MSH3 status can regulate the DNA damage response and extent of apoptosis induced by chemotherapy. The ability of MSH3 to regulate chemosensitivity was independent of MLH1 status. PCI-24781-mediated impairment of HR enhanced oxaliplatin sensitivity, suggesting that reduced DSB repair capacity may be contributory.

## Introduction

The DNA mismatch repair (MMR) system is composed of proteins (MLH1, MSH2, MSH3, MSH6, PMS2) whose function is to correct base-base mispairs introduced into short, tandemly repeated sequences, termed microsatellites, during DNA synthesis to maintain genomic stability [1]. MMR proteins interact as heterodimers and when a mismatch is detected, a series of steps occur that include the association of MSH2 with either MSH6 or MSH3 to form MutSα or MutSβ complexes, respectively [2]. Either MutSα, a MSH2/MSH6 heterodimer with higher affinity for recognizing single base mismatches, or MutSβ, an MSH2/MSH3 heterodimer with higher affinity for recognizing 2–13-bp insertion-deletion loops, binds to the DNA mismatch [3]. Similarly, interaction of MLH1 with PMS2 forms the MutLα complex that forms a ternary complex with a MutS heterodimer that binds to DNA mismatches and coordinates excision of the DNA mispair [4]. Excision of the mismatch is subsequently followed by re-synthesis and repeat ligation of the DNA strand [4].

Deficient DNA MMR is found in approximately 15% of human colorectal cancers (CRCs) that display a distinct tumor phenotype [5]. Consistent evidence indicates that deficient MMR colon cancers have a more favorable clinical outcome as compared to proficient MMR tumors [6]. Furthermore, these tumors appear to lack benefit from 5-fluorouracil as adjuvant chemotherapy [7], [8]. The molecular fingerprint of deficient MMR is the presence of microsatellite instability (MSI) [1]. MSI is characterized by increased mutations in microsatellites that arise due to germline mutations in MMR genes (*MLH1, MSH2, MSH6* and *PMS2*) causing Lynch Syndrome, or more commonly as a consequence of *MLH1* hypermethylation in association with the CpG island methylator phenotype (CIMP) in sporadic CRCs [2].

The *MSH3* gene undergoes frequent (43–52%) somatic mutation in MMR-deficient CRCs [9], [10], [11], [12], [13]; however, germline mutations in *MSH3* have not been identified. Among CRCs examined as part of the Cancer Genome Atlas project, *MSH3* mutations were detected in 40% of hypermutated tumors of which three quarters showed MSI [14]. Loss of MSH3 protein expression follows biallelic frameshift mutation at the (*A*)_8_ coding mononucleotide repeat in exon 7 of *MSH3* [9], [15]. The *MSH3* gene, located on chromosome 5q11–q12 [16], encodes the MSH3 protein that has a partially redundant function with MSH6 [3]. *MSH3*-deficient mice develop late onset MSI-positive gastrointestinal cancer [17], suggesting that *MSH3* deficiency can contribute to tumor initiation [9]. While MSH3 appears to play a role in CRC development, its ability to regulate chemosensitivity remains unproven. Evidence indicates that the MSH2/MSH3 heterodimer, known as MutSβ, is involved in the repair of toxic DNA double strand breaks (DSBs) induced by interstrand crosslink (ICLs) that are formed by agents such as cisplatin and psoralen [18]. The ability of homologous recombination (HR) to repair ICLs is dependent on MutSβ, but not on MutSα or MLH1. Recent data suggest that MSH3 is involved in the repair of oxaliplatin-induced DNA ICLs [19], [20], [21], and co-localizes to DSB lesions that were induced by laser treatment [22] or by a carcinogen [18]. Important for HR repair of DSBs are histone deacetylase (HDAC) enzymes [23], [24]. HDAC inhibitors, such as the phenyl hydroxamic acid (PCI-24781), have emerged as a class of therapeutic agents with broad anti-tumor activity. Recent data showing synergy of HDAC inhibitors with ionizing radiation and other DNA-damaging agents suggest that HDAC inhibitors may act, in part, by inhibiting DNA repair.

We hypothesized that MSH3 status, independent of MLH1, can regulate response to DSB breaks induced by the irinotecan metabolite, SN-38, and the ICL-inducing agent oxaliplatin. Furthermore, we tested the hypothesis that the HDAC inhibitor, PCI-24781, inhibits HR to impair DSB repair that may result in enhanced cytotoxicity. We provide evidence that MSH3 can modulate the extent of DNA damage and the cytotoxicity of anti-cancer drugs, and demonstrate that *MSH3* deficiency can suppress HR that repairs DSBs.

## Results

### Effect of MSH3 on cytotoxicity of anti-cancer drugs

**Figure 1:**
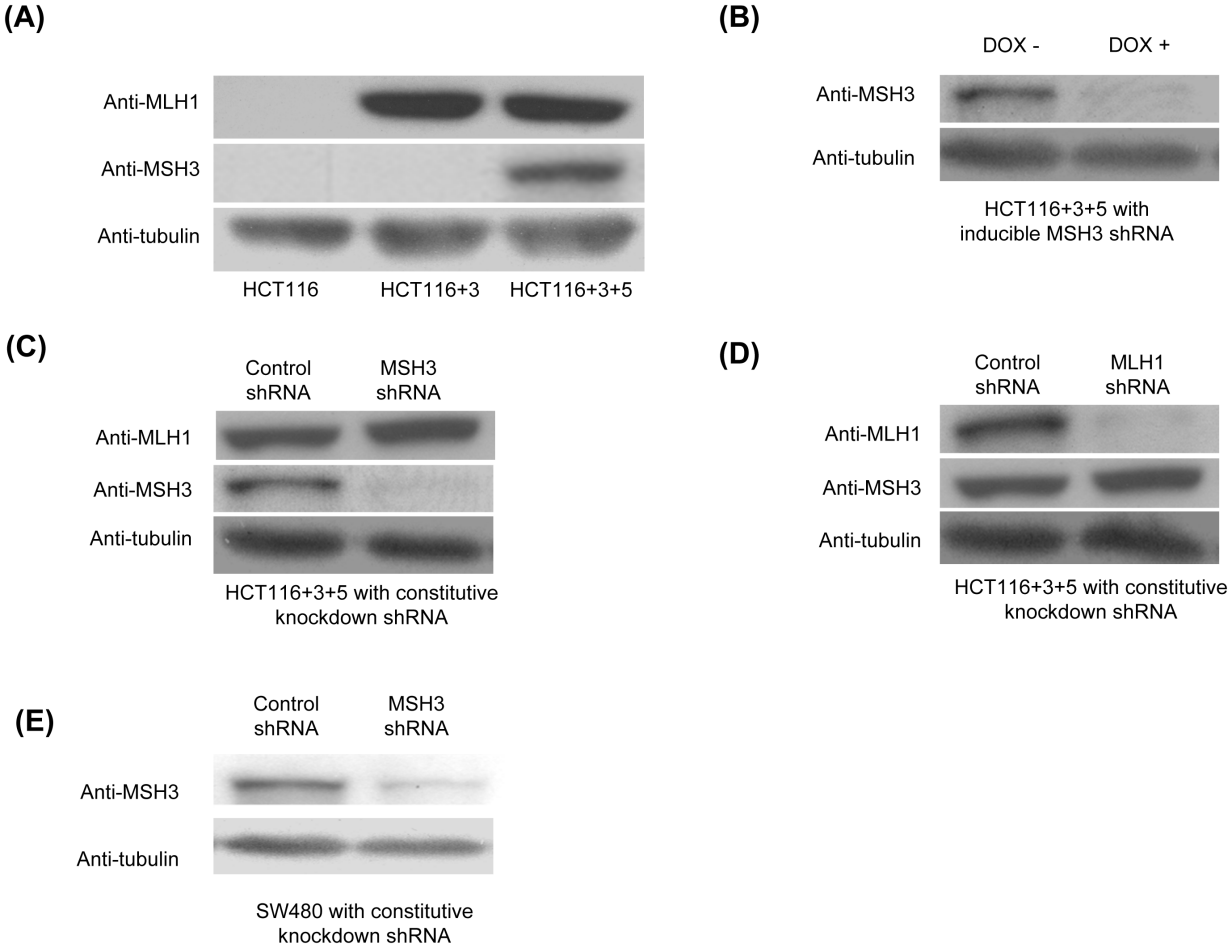
MSH3 protein expression in HCT116-derived clones and SW480 cells. A, Western blot analysis of MSH3, MLH1, and tubulin in HCT116, HCT116+3, and HCT116+3+5 cells. B, MSH3 expression is controlled by a Tet-on shRNA system in HCT116+3+5 cells cultured in medium with and without 1 µg/ml doxycycline. C, D MSH3 or MLH1 expression was suppressed using constitutive *MSH3* or *MLH1* knockdown by shRNA in HCT116+3+5 cells. E, MSH3 expression was also suppressed using constitutive *MSH3* shRNA in SW480 cells.

**Figure 2:**
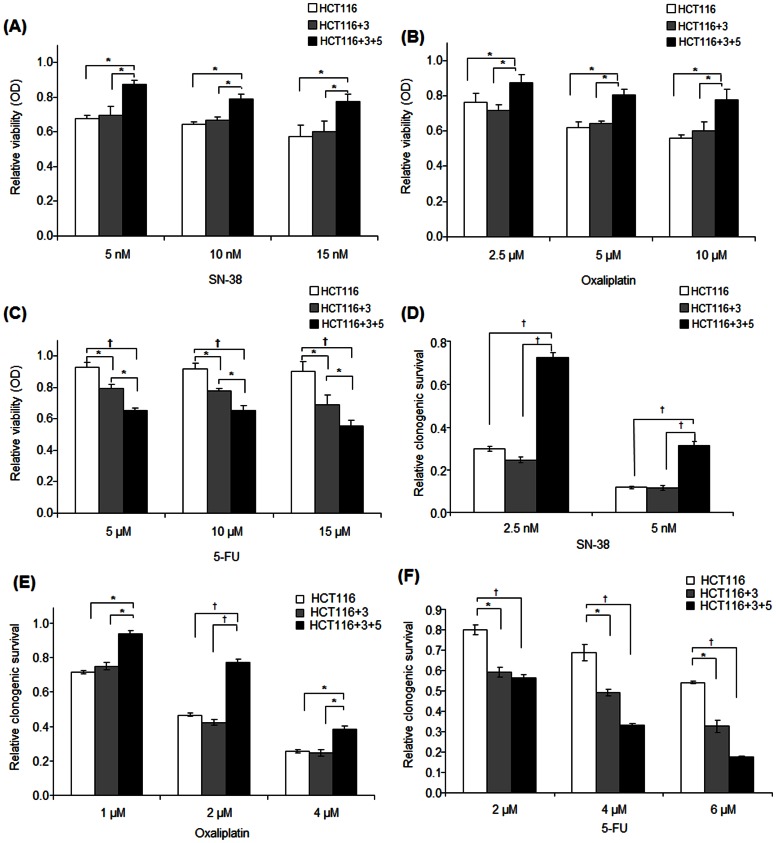
*MSH3*-deficient (vs proficient) cells are more sensitive to SN-38 and oxaliplatin in contrast to 5-FU. A–C, MTS assay in HCT116 (*MLH1−/MSH3−*), HCT116+3 (*MLH1+/MSH3−*), and HCT116+3+5 (*MLH1+/MSH3+*) cells treated with SN-38 (A), oxaliplatin (B) or 5-FU (C). D–F, Clonogenic survival of these same cell lines treated for 48 h with SN-38 (D), oxaliplatin (E), or 5-FU (F). Data are presented as mean ± standard errors *vs* controls for at least 3 independent experiments. Statistical significance was determined by a two-sided Student's t test (* P<0.05; † P<0.01).

We determined the effect of MSH3 expression upon cell viability in response to anti-cancer drugs including SN-38, the primary metabolite of the topoisomerase I inhibitor irinotecan, the alkylating and DNA intercalating agent oxaliplatin, and the nucleoside analogue 5-fluorouracil (5-FU). The isogenic cell lines used in this study and the status of MSH3 and MLH1 expression are shown in Fig. 1A. HCT116+3+5 cells (*MSH3+/MLH1+*) were shown to be the least sensitive to treatment with SN-38 or oxaliplatin compared to HCT116+3 (*MSH3−/MLH1+*) and parental HCT116 cells (*MSH3−/MLH1−*), and the extent of growth inhibition was similar among the latter two cell lines (Fig. 2A, B). In contrast, HCT116+3+5 cells were the most sensitive to 5-FU, HCT116+3 cells were intermediate, and HCT116 cells were least sensitive (Fig. 2C). The chemosensitivity of these cell lines was also studied in a long-term clonogenic survival assay with highly concordant results for treatment with SN-38 or oxaliplatin (Fig. 2D, E) and for 5-FU (Fig. 2F).

**Figure 3:**
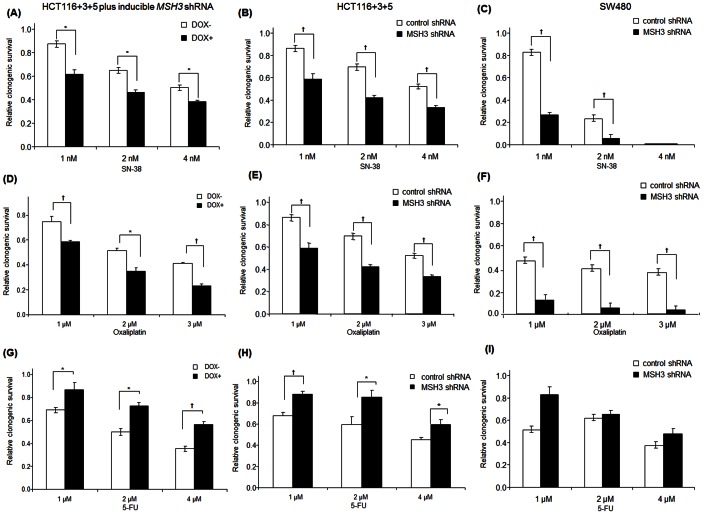
Clonogenic survival in relation to *MSH3* knockdown in HCT116+3+5 and SW480 cells treated with SN-38, oxaliplatin or 5-FU. A, D, The effect of suppression of *MSH3*, using an inducible shRNA system (doxycycline 1 µg/ml, DOX+), upon chemosensitivity of cell lines to treatment with SN-38 (A) or oxaliplatin (D) [48 h] relative to control cells (DOX-). B,C,E,F, HCT116+3+5 and SW480 cells lines with constitutive knockdown of *MSH3* by shRNA were treated with SN-38 (B, C) or oxaliplatin (E, F) and clonogenic survival was determined relative to cells with control shRNA. G,H,I, HCT116+3+5 cells with *MSH3* suppression (DOX+) or gene knockdown using shRNA were treated with 5-FU for 48 h and clonogenic survival was determined compared to control cells (DOX- or non targeted shRNA). Data are presented as mean ± standard errors *vs* control cells from at least 3 independent experiments. Statistical significance was determined by a two-sided Student's t test (* P<0.05; † P<0.01).

To confirm the effect of MSH3 status upon clonogenic survival, we utilized a Tet-on system and also generated cells with constitutive knockdown of *MSH3* by lentiviral shRNA (Fig. 1B,C). In HCT116+3+5 cells treated with SN-38, suppression of *MSH3* (DOX+) significantly sensitized the cells to SN-38 compared to control cells (DOX-), as indicated by fewer surviving colonies (Fig. 3A). HCT116+3+5 and SW480 cells with constitutive knockdown of *MSH3* were also significantly sensitized to SN-38 (Fig. 3B, C). Similarly, HCT116+3+5 cells with suppression of *MSH3* (DOX+) or HCT116+3+5 and SW480 cells with constitutive knockdown of *MSH3* were rendered sensitive to oxaliplatin compared to control cells (DOX- or non-targeted shRNA) (Fig. 3D, E, F). In contrast, HCT116+3+5 cells with *MSH3* suppression (DOX+) or HCT116+3+5 and SW480 cells with constitutive knockdown of *MSH3* were rendered resistant to 5-FU compared to control cells (DOX- or non targeted shRNA) (Fig. 3G, H, I). Together, these data indicate that MSH3 confers resistance to SN-38 and oxaliplatin, but not to 5-FU, treatment in cells with or without expression of MLH1.

### Effect of MSH3 status upon chemotherapy-induced DNA damage and apoptosis

**Figure 4:**
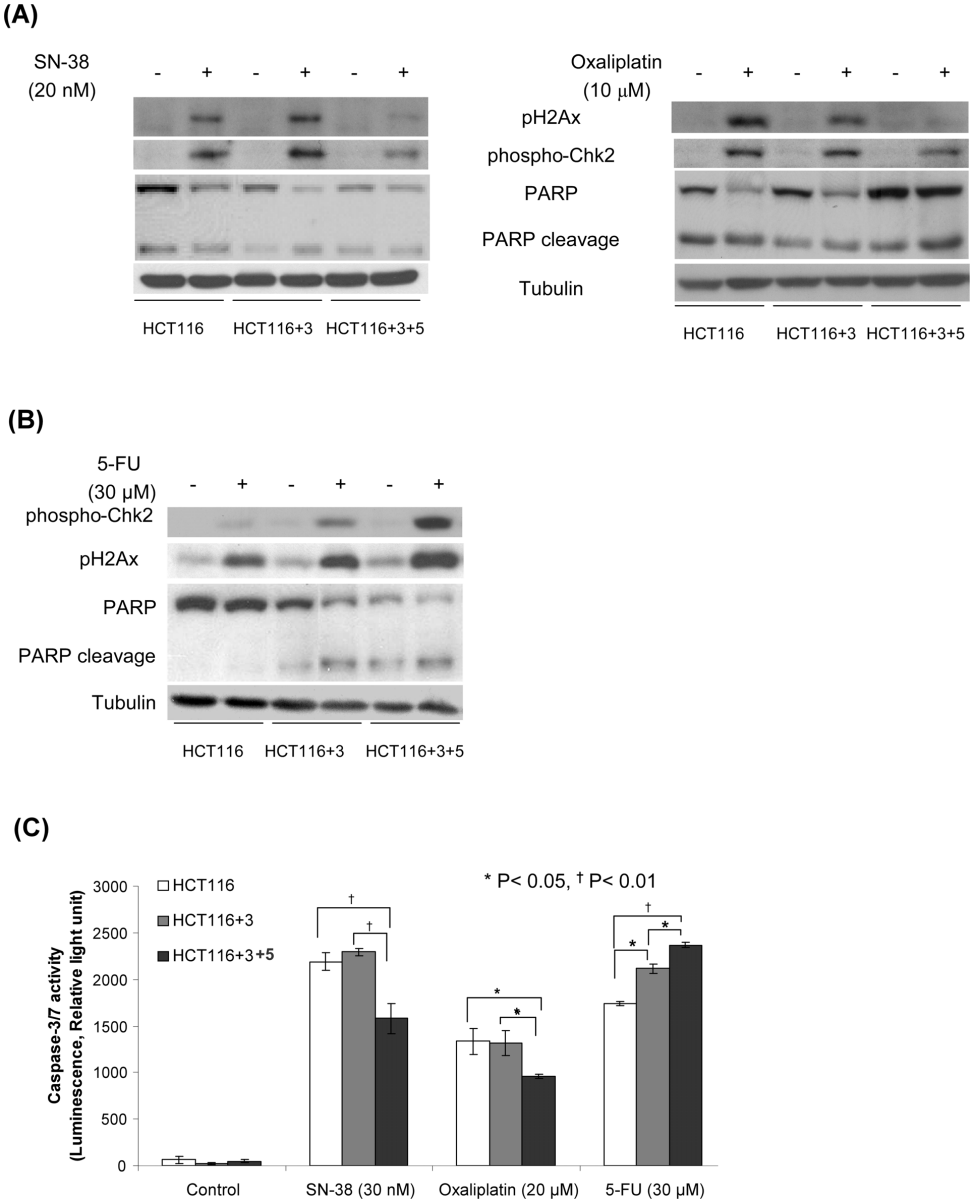
Effect of chemotherapeutic agents on DNA damage and apoptosis in HCT116, HCT116+3 and HCT116+3+5 cells. A, B, Analysis of pH2AX and pChk2 expression and PARP cleavage in HCT116, HCT116+3, and HCT116+3+5 cells treated with 5-FU, SN-38 or oxaliplatin for 48 h and compared to untreated control cells. C, Analysis of caspase-3/7 activity in HCT116, HCT116+3 and HCT116+3+5 cells treated with 5-FU, SN-38 or oxaliplatin (48 h). Data are shown as mean ± standard errors from 3 independent experiments. Statistical significance was determined by a two-sided Student's t test (* P<0.05; † P<0.01).

To determine whether MSH3 is involved in DNA DSB repair, we determined the effect of anti-cancer drug treatment upon expression of the DSBs markers, phosphorylated histone H2AX (pH2AX) and Checkpoint kinase 2 (pChk2) [25]. Induction of DSBs is known to trigger the phosphorylation of histone H2AX that is mediated by ATM which also mediates Chk2 activation. Chk2 regulates the cell cycle response to DNA damage [26]. After its dissociation from chromatin, Chk2 can phosphorylate substrates including Cdc25 enzymes that are responsible for cell cycle arrest [27] and the transcription factor, E2F-1 that promotes apoptosis [26]. Treatment with SN-38 or oxaliplatin significantly increased the level of pChk2 and pH2AX expression in *MSH3*-deficient cells (HCT116, HCT116+3) to a greater extent than in *MSH3*-proficient cells (HCT116+3+5) (Fig. 4A). With 5-FU treatment, an opposite result was observed whereby *MSH3*-deficient cells showed decreased susceptibility to DNA damage indicated by reduced pChk2 and pH2AX levels. In response to 5-FU, DNA damage markers were lowest in cells deficient in *MLH1* and *MSH3* (HCT116), intermediate in cells with only *MLH1* (HCT116+3), and highest in HCT116+3+5 cells proficient for *MLH1* and *MSH3* (Fig. 4B). A similar effect of MSH3 status on DNA damage and cell death was observed. Specifically, *MSH3*-deficient *vs* -proficient cells were sensitized to apoptosis induction by SN-38 or oxaliplatin, but not 5-FU, as indicated by increased PARP cleavage (Poly ADP ribose polymerase) and caspase-3/7 activation (Fig. 4A–C). This finding suggests that SN-38 and oxaliplatin may overwhelm the cellular DNA repair capacity, thus leading to cell death. In contrast, 5-FU treatment of *MSH3* knockdown cells showed less DNA damage compared to the other drugs, suggesting that DNA repair processes may rescue these cells from apoptosis as shown by reduced cleavage of PARP and caspase-3/7, compared to cells with intact MSH3 (Fig. 4B, C).

**Figure 5:**
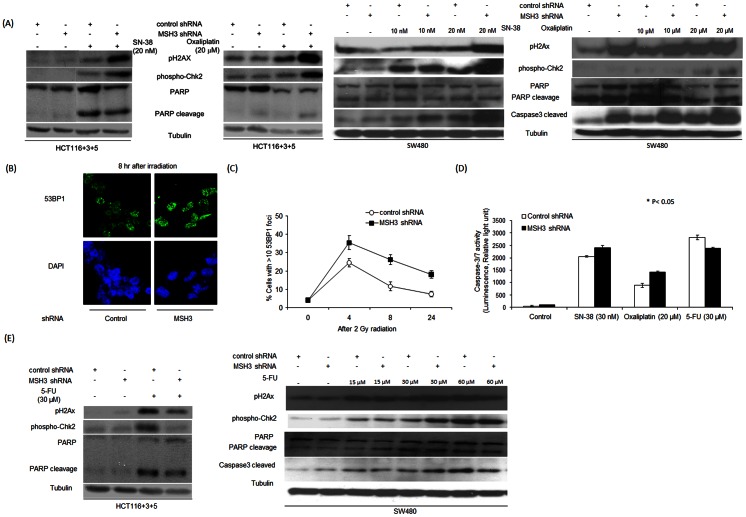
Effect of chemotherapeutic agents on DNA damage and apoptosis in *MSH3*-proficient and –deficient cells. A, Analysis of pH2AX and pChk2 expression and PARP cleavage in *MSH3*–deficient HCT116+3+5 cells (left) and SW480 cells (right) with constitutive shRNA knockdown of *MSH3*. Cells were treated with SN-38 or oxaliplatin for 48h. B, C, Immunofluorescence staining to detect the formation of 53BP1 nuclear foci in irradiated (2Gy) HCT116+3+5 cells with control shRNA (left) versus shRNA suppression of *MSH3* (right). The percentage of cells with >10 53BP1 nuclear foci was determined at the indicated time-points. At least 100 total cells were counted in each slide. D, Analysis of caspase-3/7 activity in HCT116+3+5 cells with and without *MSH3* knockdown that were treated with 5-FU, SN-38 or oxaliplatin (48 h). E, Analysis of pH2AX and pChk2 expression and PARP cleavage in 5-FU-treated (48h) HCT116+3+5 cells (left) that are *MSH3*–deficient and in SW480 cells (right) with constitutive shRNA knockdown of MSH3. Data represent mean ± standard errors from 3 independent experiments. Statistical significance was determined by a two-sided Student's t test (* P<0.05; † P<0.01).

We also performed gene knockdown experiments in HCT116+3+5 and SW480 cells (Fig. 1C,E) to isolate the impact of *MSH3* deficiency. Treatment with SN-38 or oxaliplatin increased pH2AX, pChk2 and PARP cleavage in cells with constitutive knockdown of *MSH3 vs* control (Fig. 5A). To further study the differential effect of MSH3 status on the DNA damage response, we examined the formation of 53BP1 nuclear foci that are recruited to sites of DNA DSBs that occur after radiation [28]. In *MSH3* knockdown and control cells treated with gamma irradiation, suppression of *MSH3* was shown to significantly increase the percentage of cells containing >10 53BP1 foci compared to control shRNA cells (Fig. 5B, C). *MSH3* knockdown cells treated with 5-FU showed less DNA damage compared to the other drugs, suggesting that DNA repair processes may rescue these cells from apoptosis as shown by reduced cleavage of PARP and caspase-3/7, compared to *MSH3*-proficient cells (Fig. 5D, E).

### Impact of MSH3 on chemosensitivity is independent of MLH1 expression

**Figure 6:**
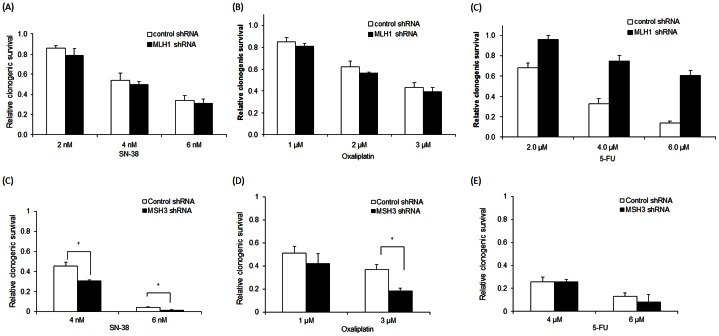
Clonogenic survival after drug treatment of HCT116+3+5 cells with *MLH1* knockdown or *MLH1*-deficient SW48 cells with *MSH3* knockdown. A, B, HCT116+3+5 cells with constitutive knockdown of *MLH1* were treated for 48 h with SN-38 (A), oxaliplatin (B), or 5-FU (C) and the number of surviving colonies was determined and compared to control cells. D–F, *MLH1*-deficient SW48 with constitutive knockdown of *MSH3* by shRNA were treated with SN-38 (D), oxaliplatin (E), or 5-FU (F). The number of surviving colonies was then determined and compared to the control cells. Data are presented as mean ± standard errors from at least 3 independent experiments. Statistical significance was determined by a two-sided Student's t test (* P<0.05; ^†^ P<0.01).

To determine the potential impact of MLH1 upon chemosensitivity in HCT116+3+5 cells (*MSH3+/MLH1+*), we suppressed *MLH1* by shRNA (Fig. 1D) and determined their sensitivity to SN-38, oxaliplatin, or 5-FU compared to cells transfected with a non targeting shRNA. In HCT116+3+5 cells with *MLH1* knockdown, the cytotoxic effects of SN-38 or oxaliplatin were similarly enhanced as seen in cells with intact MLH1 (Fig. 6A, B), but 5-FU-related cytotoxicity was significantly attenuated (Fig. 6C). In MLH1 expressing HCT116+3 cells, 5-FU-induced cytotoxicity was significantly increased compared to *MLH1*-deficient HCT116 cells, as shown in prior studies [29], and the cytotoxic effects of SN-38 and oxaliplatin were unchanged. We also studied the effect of *MSH3* knockdown in an MMR-deficient background by using the SW48 CRC cell line that is deficient in *MLH1*. In SW48 cells, the enhanced cytotoxic effects of SN-38 and oxaliplatin were maintained after *MSH3* knockdown whereas no difference was seen for 5-FU compared to results in HCT116+3+5 cells with *MLH1* knockdown (Fig. 6D–F).

### MSH3 Regulates Response to DNA Damage

**Figure 7:**
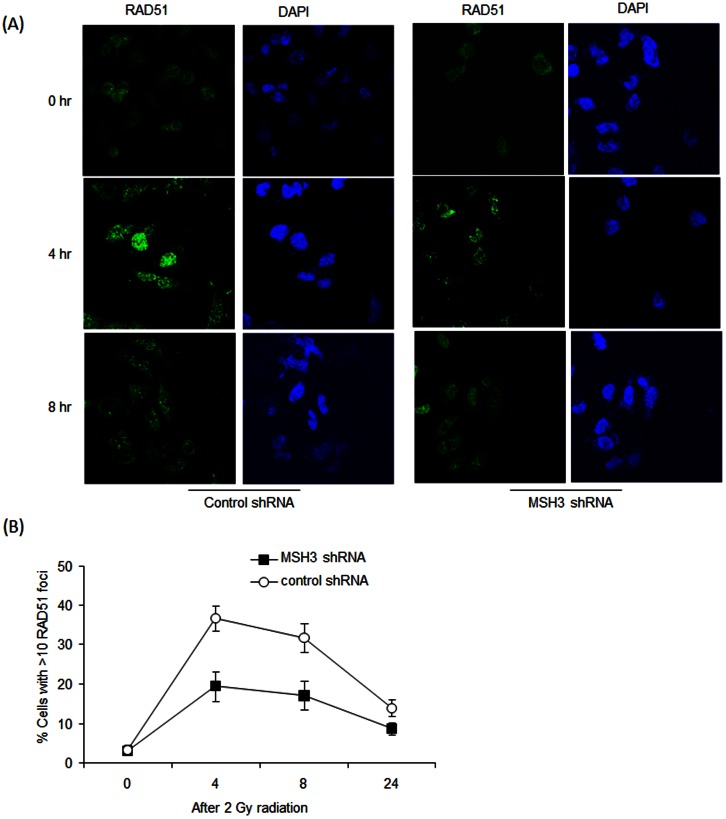
Immunofluorescence staining for RAD51 nuclear foci. A, B, HCT116+3+5 cells with control or *MSH3* shRNA were irradiated (2 Gy) and then probed with an anti-RAD51 antibody. The percentage of cells with >10 RAD51 nuclear foci in *MSH3* knockdown *vs* control cells was determined at the indicated time points. Representative images of RAD51 staining are shown in (A), and the quantification of RAD51 nuclear foci is shown in (B). Error bars represent standard errors from 3 independent experiments.

**Figure 8:**
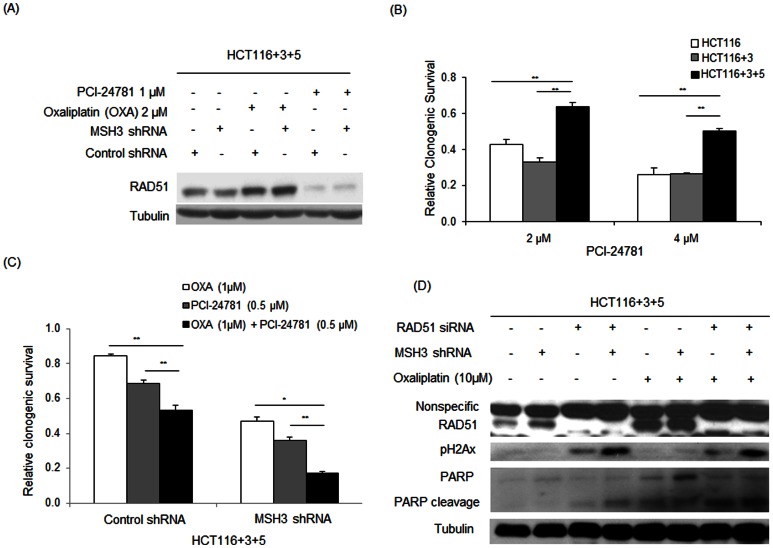
HDAC inhibitor, PCI-24781, inhibits RAD51 expression and enhances sensitivity to oxaliplatin in *MSH3*-deficient HCT116+3+5 cells. A, Effect of PCI-24781 or oxaliplatin treatment (both, 24 h) on the level of RAD51 protein expression in HCT116+3+5 cells with *MSH3* knockdown or control shRNA. B, Relative clonogenic survival of HCT116 (*MLH1−/MSH3−*), HCT116+3 (*MLH1+/MSH3−*), and HCT116+3+5 (*MLH1+/MSH3+*) treated with PCI-24781. C, Effect of PCI-24781 alone or combined with oxaliplatin on the relative clonogenic survival in HCT116+3+5 cells with *MSH3* knockdown versus control shRNA. D, Effect of oxaliplatin treatment (48 h) on the expression levels of pH2AX and cleaved PARP in HCT116+3+5 cells with knockdown of *RAD51* using siRNA or *MSH3* by shRNA. Data are represented as mean ± standard errors from 3 independent experiments. Statistical significance was determined by a two-sided Student's t test (* P<0.05; ^**^ P<0.01).

To determine if MSH3 can regulate HR, we examined the recruitment of nuclear Rad51 to sites of DNA damage by immunofluorescence staining and confocal microscopy. Treatment of *MSH3*-proficient cells with gamma irradiation (2 Gy for up to 24 hr) was shown to increase the formation of Rad51 foci. However, radiation-induced formation of Rad51 foci was significantly attenuated in *MSH3*-knockdown *vs MSH3* intact cells (Fig. 7A, B), suggesting that defective HR repair may contribute to the increased sensitivity of *MSH3*-deficient cells to radiation. In an effort to exploit the impairment in HR mediated by MSH3, we utilized the HDAC inhibitor, PCI-24781, that is a known to inhibit HR and reduce RAD51 expression [30]. We found that PCI-24781 treatment reduced RAD51 expression in *MSH3*-proficient, HCT116+3+5 cells (Fig. 8A). Furthermore, *MSH3*-deficient *vs* proficient cells displayed increased sensitivity to PCI-24781 as shown in a clonogenic survival assay (Fig. 8B). To exploit this effect, *MSH3*-deficient and –proficient cells were treated with PCI-24781, oxaliplatin, or their combination. PCI-24781 treatment was shown to significantly enhance oxaliplatin-mediated cytotoxicity (Fig. 8C), suggesting that MSH3 plays a role in the regulation of HR repair. To confirm the importance of HR in the ability of MSH3 to regulate chemosensitivity, we suppressed *RAD51* using siRNA in HCT116+3+5 cells in the presence or absence of *MSH3* shRNA and exposed the cells to oxaliplatin. In *MSH3*-deficient cells treated with oxaliplatin, knockdown of *RAD51* increased expression of the DNA DSBs markers, pHA2X and cleaved PARP (Fig. 8D).

## Discussion

Detection of a nucleotide mismatch results in the formation of heterodimeric complexes involving MSH2 and either MSH6 (MutSα) or MSH3 (MutSβ) [3]. Recent data indicate that MSH3, the recognition component of MutSß, plays an important role in recognizing DNA damage, especially ICLs that are formed by platinum compounds [19], [21]. In this study, we determined the impact of MSH3 on cytotoxicity induced by anti-cancer drugs used for CRC treatment. Using isogenic colon cancer cell lines in which *MSH3* expression was suppressed by a Tet-on system or by constitutive knockdown, we found that the cytotoxic effects of SN-38 and oxaliplatin were dependent upon MSH3 status. *MSH3*-deficient cells were more sensitive to SN-38 and oxaliplatin compared to *MSH3*-proficient cells that was unrelated to MLH1 status. Furthermore, *MSH3*-deficient cells were more susceptible to radiation-induced DNA DSBs, as shown by higher levels of pH2AX, pChk2 and 53BP1 (marker of nonhomologous end joining), and apoptosis after oxaliplatin or SN-38 treatment. Accordingly, *MSH3*-deficient cells may become overwhelmed by the extent of DNA damage and undergo apoptosis whereas *MSH3* proficiency provides protection from DSBs. In support of our data in isogenic HCT116 cells, we also generated SW480 colon cancer cells with suppression of MSH3. Similar results were found for the effect of MSH3 status upon chemosensitivity, suggesting that our results can be generalized.

In contrast to SN-38 or oxaliplatin, *MSH3*-deficient *vs* proficient cells showed reduced sensitivity to 5-FU coincident with reduced expression of markers of DNA DSBs. However, *MLH1* knockdown confered resistance to 5-FU in the presence of MSH3, indicating that MLH1 contributes to 5-FU resistance in *MSH3*-deficient cells which was not the case for SN-38 or oxaliplatin. 5-FU is incorporated into DNA and is recognized by the DNA MMR system [31]. MSH2-MSH3 (MutSβ) and MSH2-MSH6 (MutSα) complexes recognize 5-FU incorporated into DNA, and the extent of 5-FU-induced cytotoxicity is greatest when both complexes are intact [32]. It is also possible that MSH2-MSH3 recognition of 5-FU incorporated into DNA might trigger MMR-independent repair mechanisms in addition to MMR [33], [34], [35].

Our study findings have implications for the treatment of CRC depending upon MSH3 status in the tumor. Importantly, experiments were conducted using clinically relevant doses of chemotherapy drugs. The *MSH3* gene undergoes frequent (40%) somatic mutation in hypermutated CRCs [14], the majority of which are MMR-deficient [9], [10], [11], [14]. In MLH1-deficient human colon cancers, loss of MSH3 protein expression was found in 37–48% of tumors [11], [36]. Reduced or heterogeneous expression of MSH3 proteins is associated with elevated microsatellite alterations at selected tetranucleotide repeats (EMAST) that occurs during MSI, but is not associated with major defects in DNA MMR [9], [37]. While the predictive impact of MSH3 or EMAST for therapeutic response to anti-cancer drugs in human CRCs awaits further study, our preclinical data suggest that human tumors with reduced MSH3 expression would be expected to demonstrate resistance to 5-FU, yet show increased sensitivity to oxaliplatin and irinotecan. Since sporadic CRCs with frameshift *MSH3* mutations will generally be MMR deficient due to epigenetic inactivation of *MLH1*, we confirmed that suppression of *MSH3* in cells deficient in *MLH1* can regulate chemosensitivity.

Consistent with prior studies in cell lines [29], [38] and tumor xenografts [39], we found that *MLH1*-deficient HCT116 cells were resistant to 5-FU compared to *MLH1*-proficient HCT116+3 cells. Studies have shown that patients with MSI CRCs show a lack of survival benefit from 5-FU as adjuvant therapy, including data from randomized clinical trials with untreated control arms [7], [8] and a meta-analysis [6]. In contrast to 5-FU, we found that HCT116+3 cells (+ch 3 restores *MLH1*) [9] showed sensitivity to oxaliplatin and SN-38 in contrast to parental HCT116 cells, consistent with data indicating that MMR proteins do not recognize oxaliplatin-related adducts [20], [40]. Consistent with our data for SN-38, MSI *vs* MSS colon cancer cells and tumor xenografts show increased sensitivity to irinotecan [41], [42], [43]. However, data are conflicting for irinotecan benefit in patients with MSI colon cancers treated with irinotecan plus 5-FU *vs* 5-FU alone [44], [45]. An important determinant of SN-38/irinotecan sensitivity is frequent mutations in the *MRE11A* and *hRAD50* genes in MSI cell lines and tumors that control repair of DNA DSBs [41], [46], [47]. Mutations in these genes have been shown to confer sensitivity to camptothecins compared to cells with wild-type copies [41].

Tumor cells utilize their DNA repair capacity to prevent an accumulation of lethal DNA DSBs from cytotoxic chemotherapy or radiation [40]. Studies in yeast indicate that MSH3 can enable HR to repair DSBs [48]. After exposure to radiation, RAD51 rapidly forms a complex with BRCA2 and other proteins that stimulate RAD51-mediated strand exchange and the assembly of subnuclear foci characteristic of HR [49]. In addition to repair of radiation-induced DNA damage, RAD51 is involved in the repair of DNA DSBs produced by cisplatin, camptothecins and inhibitors of PARP [50], [51], [52]. In our study, the percentage of cells with >10 RAD51 foci was significantly decreased after radiation in *MSH3*-defieicnt *vs* -proficient cells, suggesting that MSH3 can regulate HR. In a recent study, *MSH3*-deficiency resulted in EMAST and pathway analysis revealed overexpression of proteins involved in DSB repair (MRE11 and RAD50) and apoptosis in HCT116 and HCT116+ch3 cells [53].

We utilized the HDAC inhibitor PCI-24781 that is known to inhibit HR [29]. PCI-24781 was shown to markedly decrease the level of RAD51 expression that was associated with the ability of this drug to overcome MSH3-mediated resistance to oxaliplatin. PCI-24781 significantly enhanced the cytotoxic effect of oxaliplatin in *MSH3*-proficient cells to a comparable extent as in *MSH3*-deficient cells. In support of these data, knockdown of RAD51 in oxaliplatin-treated cells was shown to increase the expression of DNA damage markers.

In summary, *MSH3* deficiency confers increased sensitivity to oxaliplatin and SN-38, but not 5-FU, in association with increased DNA DSBs in isogenic colon cancer cell lines. Higher levels of drug-induced apoptosis in *MSH3*-deficient *vs* proficient cells may be related, in part, to an impaired ability to repair DNA DSBs. Irradiated MSH3-deficient cells showed reduced numbers of RAD51 foci indicating impaired HR. PCI-24781, an HDAC inhibitor that interferes with HR, produced a marked reduction in RAD51 expression, and both this drug and RAD51 knockdown were shown to confer sensitivity to oxaliplatin. While independent of MSH3 status, sensitivity to 5-FU was dependent upon MLH1. Taken together, our results suggest a functional role for MSH3 in the DNA damage response and potentially in the process of HR that may contribute its ability to regulate chemosensitivity. Importantly, MSH3 was shown to mediate chemosensitivity independent of *MLH1* status and therefore, does not require participation of the canonical MMR pathway [32]. These data have clinical implications in that they may influence the selection of chemotherapeutic agents based upon tumor MSH3 status, and provide a rationale for the evaluation of MSH3 as a predictive biomarker in CRC patients.

## Materials and Methods

### Reagents

Oxaliplatin, 5-FU and SN-38 were purchased from Sigma-Aldrich (St. Louis, MO) and PCI-24781 from Selleckchem Chemicals (Houston, TX). Drugs were dissolved in DMSO to produce a 200 mmol/L stock solution that was aliquoted and stored at −20°C.

### Cell lines and cell culture

The human colon cancer cell lines HCT116, HCT116+chromosome 3 (HCT116+3), and HCT116+chromosome 3+chromosome 5 (HCT116+3+5) [gift of C.R. Boland, Baylor Univ., Dallas, TX] have been described previously [9], [54] (Fig. 1). Cells were grown in Iscove's Modified Dulbecco's Medium (Invitrogen, Carlsbad, CA) with 10% fetal bovine serum. We also utilized 293T cells that were grown in DMEM (Sigma-Aldrich), SW48 and SW480 cells that were grown in RPMI (Invitrogen). SW480 cells were both MLH1 and MSH3 proficient and SW48 cells were MLH1-deficient and MSH3-proficient. Cell lines were authenticated every 6 months using a panel of genetic and epigenetic markers. We analyzed the expression of MSH3 and MLH1 proteins in HCT116, HCT116+3, and HCT116+3+5 cells (Fig. 1A). HCT116 cells are both MLH1 and MSH3 deficient whereas HCT116+3 are only MSH3 deficient since *MLH1* was restored by transfer of a copy of chromosome 3. HCT116 and HCT116+3 cells lack MSH3 expression consistent with their harboring a homozygous frameshift mutation in a mononucleotide repeat in exon 7 of *MSH3* [9]. HCT116+3+5 are generated from MSH3-deficient HCT116+3 cells by transfer of a copy of chromosome 5 that renders them both *MLH1*- and *MSH3*-proficient.

### Lentiviral short hairpin RNA

Target sequences for *MSH3, MLH1* and a control sequence were selected. MSH3 protein expression was regulated thorough short hairpin RNA (shRNA) expression using an inducible Tet-on system. HCT116+3+5 cells were stably transfected with a tetracycline-regulated lentiviral vector, pTRIPZ (Open Biosystem, Huntsville, AL) that encodes shRNA against *MSH3*. The control shRNA sequence was CAACAAGATGAAGAGCACCAA (Sigma), and the targeting sequences for *MSH3* and *MLH1* was CCGAATGCATTTGCAAGAAAT (Open Biosystem) and CACAGCATCAAACCAAGTT (Open Biosystem), respectively. To turn off expression of *MLH1* or *MSH3* shRNA, 1 µg/ml of doxycycline was added to the culture medium. Cells with constitutive *MSH3* or *MLH1* knockdown were also generated using shRNA template oligonucleotides (synthesized by the Mayo Clinic Molecular Biology Core Facility) that were ligated into an lentiviral shRNA cloning and expression vector pSIH1-H1 (System Bioscience, Mountain View, CA), as previously described [55]. Lentivirus production and transduction of target cells were performed as previously described [55]. Puromycin (2 µg/mL; Sigma) was added at 48 h post-transduction and the puromycin-resistant pool of cells was used for subsequent experiments. In cells with inducible *MSH3* shRNA, MSH3 was detected in the absence of doxycycline whereas the addition of doxycycline suppressed MSH3 expression (Fig. 1B). Cells were also generated with constitutive knockdown of *MSH3* and *MLH1* by lentiviral shRNA which inhibited expression compared to control shRNA cells (Fig. 1C,D,E).

### Small interfering RNA

RAD51 expression was suppressed using small interfering RNA (siRNA) and an irrelevant siRNA (control) (Qiagen, Valencia, CA). Transfection was performed using Lipofectamine™ RNAiMAX according to manufacturer's protocol (Invitrogen).

### 3-(4,5-dimethylthiazol-2-yl)-5-(3-carboxymethoxyphenyl)-2-(4-sulfophenyl)-2H-tetrazolium (MTS) assay

Cells were seeded into 96 well plates at a density of 4,000 cells/well in 200495l of medium. After 24 h, chemotherapeutic agents at the desired concentrations were added into each well and plates were incubated for 48 h in an incubator (5% CO_2_) at 37°C. Then, 20 µl MTS solution (Promega,) was added into each well and cells were incubated for 2 h. After this incubation, MTS absorbance was measured at 490 nm wavelength with a microplate reader.

### Clonogenic survival assay

Two hundred cells were seeded into each well of a six-well plate. After attachment, the cells were treated with drugs for 48 h and after 8–10 days, the number of colonies (colonies with >50 cells) were counted and the relative change in clonogenic survival of drug-treated versus untreated cells was determined.

### Western blot analysis

Protein samples were prepared in a lysis buffer [5 mmol/L MgCl2, 137 mmol/L KCl, 1 mmol/L EDTA, 1 mmol/L EGTA, 1% CHAPS, 10 mmol/L HEPES (pH 7.5)] containing a protease inhibitor cocktail (Sigma), as previously described [55]. Samples were normalized using nanodrop measurement (Thermo Scientific), boiled in LDS sample buffer (Invitrogen), and loaded onto 14% SDS-PAGE gel for separation of protein with electrophoretic transfer onto a polyvinylidene difluoride membrane (Bio-Rad, Hercules, CA). Membranes were probed using primary antibodies as follows: anti-human MSH3 mouse monoclonal antibody (mAb) (dilution: 1∶250, clone 52, BD Pharmingen, San Jose, CA), anti-human MLH1 mouse mAb (1∶200, G168–728, BD Pharmingen), anti-human phospho-Chk2 rabbit mAb (1∶1000, #2661, Cell Signaling Technology, Denvers, MA), anti-human phospho-Histone H2AX rabbit mAb (1∶1,000, #2577, Cell Signaling), anti-human anti-Poly(ADP-ribose) polymerase mouse mAb (PARP, 1∶5,000, #9532, Cell Signaling), anti-human cleaved caspase 3 rabbit mAb (1∶1,000, #9961, Cell Signaling), anti-human RAD51 rabbit mAb (1∶1,000, #100469, Genetex), and anti-human anti-tubulin mouse mAb (1∶10,000, clone AC-15, Sigma-Aldrich).

### Caspase-3/7 activity assay

For measurement of caspase 3/7 activity, the Caspase-Glo®/7 Assay (Promega, Madison, WI) was used according to the manufacturer's instructions. This kit is based on the cleavage of the DEVD sequence of a luminogenic substrate by caspase−3 and −7 resulting in a luminescent signal.

### Immunofluorescence

Cells were cultured on coverslips coated with poly-L-lysine and exposed to irradiation (2 Gy). Cells were fixed for 10 min with 4% paraformaldehyde, permeabilized with 0.5% Triton X-100 in PBS, and blocked in 1% fetal bovine serum. Then, cells were stained with polyclonal rabbit anti-RAD51 (H-92, Santa Cruz 1∶1000) or anti-53BP1 antibody (Cell signaling), followed by corresponding fluorescent secondary antibodies (Molecular Probes, NY). Slides were rinsed, immersed in 0.05 µg/mL 4′,6-diamidino-2-phenylindole (Invitrogen) for 5 min, rinsed again and mounted with coverslips using 10 µL Prolong Gold (Invitrogen) as the anti-fade mounting medium. Images were obtained using a Zeiss LSM 510 inverted confocal microscope with a planapochromat 63x/NA 1.4 oil immersion objective. Images were processed using Adobe Photoshop or Adobe Illustrator (Adobe Systems Inc). The frequencies of cells containing foci were determined in triplicate experiments. At least 100 nuclei were counted on each slide.
